# Neural Imaging Using Single-Photon Avalanche Diodes

**DOI:** 10.15412/J.BCN.03080103

**Published:** 2017-01

**Authors:** Mohammad Azim Karami, Misagh Ansarian

**Affiliations:** 1. Department of Electronic Engineering, School of Electrical Engineering, University of Science and Technology, Tehran, Iran.

**Keywords:** Neuroimaging, Medical imaging

## Abstract

**Introduction:**

This paper analyses the ability of single-photon avalanche diodes (SPADs) for neural imaging. The current trend in the production of SPADs moves toward the minimum dark count rate (DCR) and maximum photon detection probability (PDP). Moreover, the jitter response which is the main measurement characteristic for the timing uncertainty is progressing.

**Methods:**

The neural imaging process using SPADs can be performed by means of florescence lifetime imaging (FLIM), time correlated single-photon counting (TCSPC), positron emission tomography (PET), and single-photon emission computed tomography (SPECT).

**Results:**

This trend will result in more precise neural imaging cameras. While achieving low DCR SPADs is difficult in deep submicron technologies because of using higher doping profiles, higher PDPs are reported in green and blue part of light. Furthermore, the number of pixels integrated in the same chip is increasing with the technology progress which can result in the higher resolution of imaging.

**Conclusion:**

This study proposes implemented SPADs in Deep-submicron technologies to be used in neural imaging cameras, due to the small size pixels and higher timing accuracies.

## 1. Introduction

Different technologies have been developed for the neural imaging. Earlier technologies are called X-ray computed tomography (CT), electroencephalography, magnetic resonance imaging (MRI), magnetic resonance spectroscopy (MRS), and functional magnetic resonance (fMR) (Stoll et al.,2000; Behroozi et al., 2012). There are more recent techniques for the neural imaging such as positron emission tomography (PET) (Maquet, 2000), single photon emission computed tomography (SPECT) (Whiteside, Port, & Abramowitz, 2004), florescence lifetime imaging (FLIM) (Misgeld, & Kerschensteinerm, 2006), and time correlated single-photon counting (TCSPC) (Akerboom et al., 2012). The introduction of these techniques results in more sensitive, more reliable, and more affordable neural imaging (Rauch, & Renshaw, 1995).

Single-photon avalanche diodes (SPADs) are the single-photon detectors with the standard implementation process which are extensively used in biomedical imaging. The use of complementary metal oxide semiconductor (CMOS) technology for the SPAD production is a benefit which makes it possible for the SPADs to be integrated with complex circuits on the same chip. Moreover, using standard technology for SPAD production can lead to the production of higher number of SPADs on the same chip which results in the higher resolution of imaging.

Different technologies using single-photon detectors are reviewed in the second section of this paper, while the SPAD operational principles are presented in the third section. Moreover, the third section presents the main characteristics of SPAD and reviews the progressing parameters of them. It is shown that how the SPAD production evolution result in more precise imaging both in spatial and time domain, which can improve the neural imaging process.

## 2. Methods

**Figure 1:**
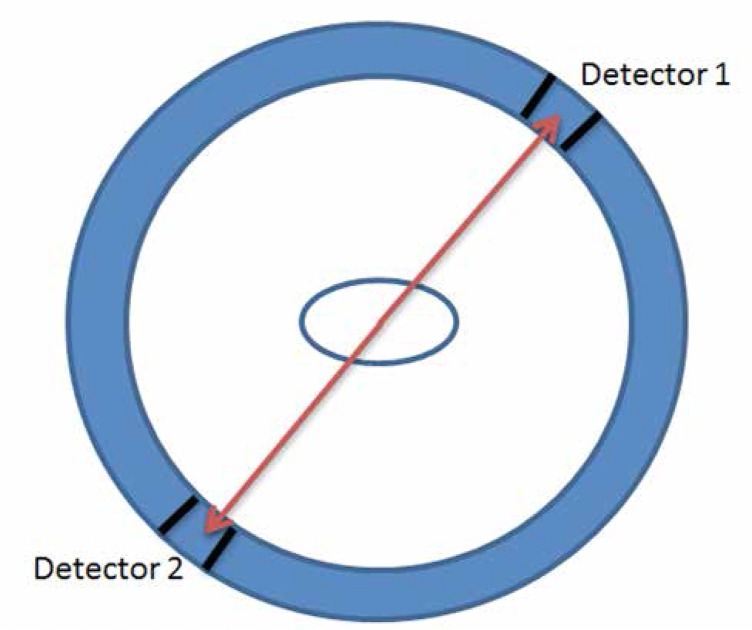
PET imaging setup consists of imaging sample at the center and the PET system. The sample is surrounded by different isolated detectors, where each detector saves the photon arrival time. The detectors have the ability to sense a single-photon arrival (Iniewski, 2009).

Positron emission tomography (PET) creates an image by measuring the electromagnetic radiation emitted by tracer molecules labeled with radioactive isotope which introduced to a living organ. Isotopes used in PET emit positrons that wipe off with atomic electrons after traveling a small distance. Each annihilation produces 2 gamma rays emitted in opposite directions and were turned to photons. The photons which are generated by the gamma ray impinging to special scintillator crystals are sensed by the detectors located around the imaging sample. Figure 1 shows the PET detection setup.

A PET scan needs special radioactive material, a positron decay, which can trace the location of the tracer radiopharmaceuticals. The radiopharmaceuticals have short lifetime and are more expensive to produce compared to the other tracers used in biomedical engineering (Iniewski, 2009). Hence, PET has to be close to the facility that makes the tracers for covering the short lifetime of the radiotracers (Phelps, 2004). Tomographic images are reconstructed through projection data provided by collinear annihilation photons that were detected by PET.

SPADs are used as the single-photon sensitive detectors to locate the photon arrival spatially and temporally (Bruschini et al., 2014). The arrival time of photons is important to form the final PET image (Braga et al., 2013).

**Figure 2:**
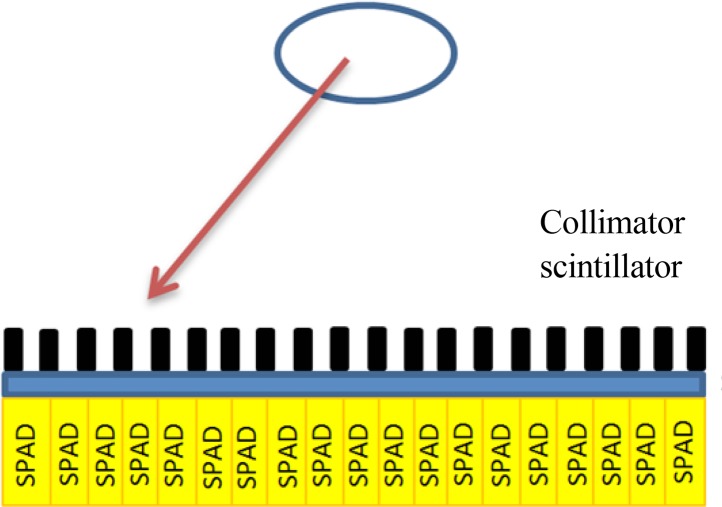
PET imaging setup consists of imaging sample at the center and the PET system. The sample is surrounded by different isolated detectors, where each detector saves the photon arrival time. The detectors have the ability to sense a single-photon arrival (Iniewski, 2009).

Unlike PET, single photon emission computed tomography (SPECT) only traces one single radiation or a general radiation, not a simultaneous double one. Hence it has a lower image resolution and is cheaper because its radioactive materials have longer lifetime. SPADs are used to make SPECT cameras too (Palubiak & Deen, 2014; Veerappan, Bruschini, & Charbon, 2012). Figure 2 shows the SPECT setup, consisting of SPADs, collimators to collect the emitted photons, scintillator crystal to convert the radioactive emission to visible photons, and some electronic readout circuits.

**Figure 3:**
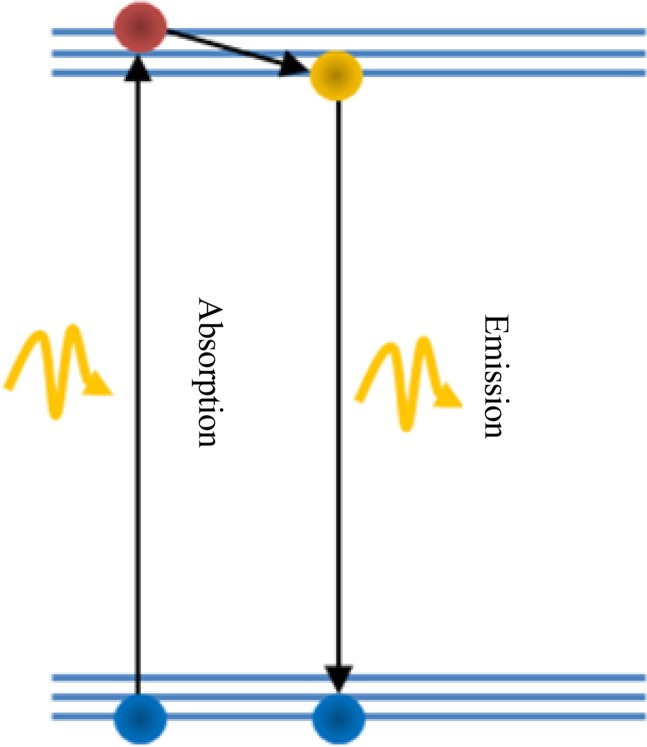
FLIM operation principle is based on molecule excitation by photon absorption and emission. The FLIM setup measures the fluorescence light emission by a sample after the photon absorption from the light source.

Figure 3 shows the main mechanism which creates the florescence lifetime imaging (FLIM) images. In this mechanism a molecule, atom, or an electron is excited using photon absorption and stays in a high energy state. After some time, the particle relaxes by emitting a different wavelength photon. The photon emission with lower frequency and higher wavelength is measured by the FLIM setup.

Using FLIM imaging, the calcium concentration changes in neural cells can be detected as a function of a given activity (Agronskaia, Tertoolen, & Gerritsen, 2004). Thus, the fluorophore molecules, previously injected into the cell, display different lifetimes depending upon the calcium concentration in the vicinity of ion channels. Several techniques exist based on FLIM, the excitation mode, or how lifetime characterization.

Using one-photon FLIM procedure, only one photon is required to force a state change in the fluorophore molecule (Agronskaia, Tertoolen, & Gerritsen, 2004). In this case, only a small shift in wavelength is observed between the excitation and response (the energy which electron loses due to thermal relaxation). Thus, filtering the excitation pulse by the measurement response is demanding. In addition, any possible scattering during the observation path induces photonic noise (usually at the same or close wavelength of fluorescence emissions) into the measurement.

**Figure 4:**
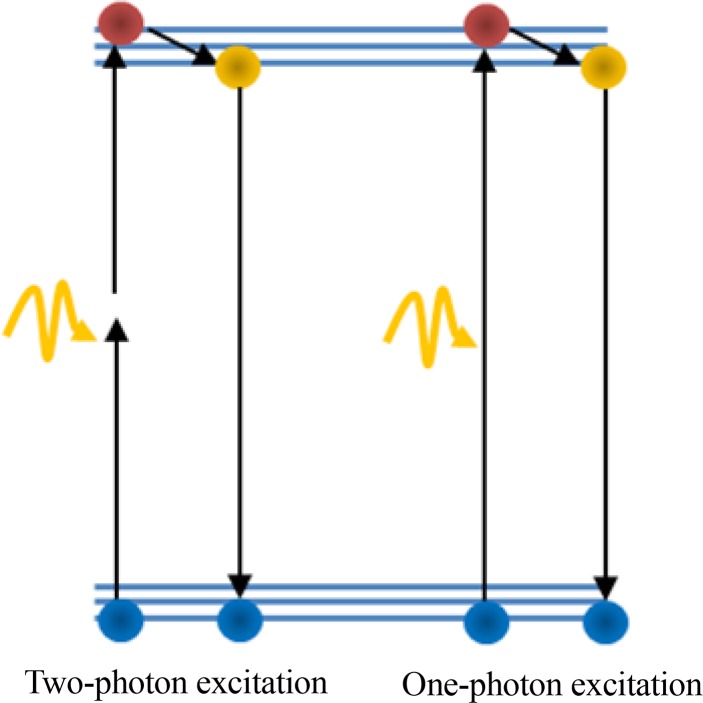
Two-photon and one-photon FLIM operation principle.

Two-photon FLIM can also be used (due to the recent advances in laser technology) to concentrate kilowatts of light power in micrometric volumes of matter. Figure 4 shows the 2-photon excitation mechanism for the 2-photon FLIM.

SPADs are used as the photosensitive element for FLIM in the photon detection with low timing uncertainty (Becker, Su, & Holub, 2011; Tyndall et al., 2011). Fluorescence lifetime imaging systems can be divided in 2 main categories: frequency domain and time domain FLIM. In frequency domain FLIM, the sample is illuminated by a modulated light source instead of a short light pulse (Spencer & Weber, 1969). In time domain FLIM, the fluorescent sample is illuminated by a pulsed laser. The laser pulses induce excited fluorophores and then the optical waveform of the fluorescent signal is monitored using either a time-gated system or time correlated single-photon counting (TCSPC) (Gersbach, 2009).

TCSPC is among the most commonly used method in time-correlated bioimaging. Several exposures are tried to piece together the statistical response of matter to the sharp light pulses. The light source repetition frequencies can vary from kHz to hundreds of MHz. From the response statistics, the molecule under observation and/or its environment, e.g. the calcium concentration, can be characterized. The fluorescence lifetime is defined as the time in which the fluorescence intensity decays to the 1/e of the initial intensity. Fluorescence lifetime is influenced by changes in the cellular environment, such as pH, or ion concentration. SPADs can be used for TCSPC (Ghioni et al., 2006; Markovic et al., 2010).

**Figure 5:**
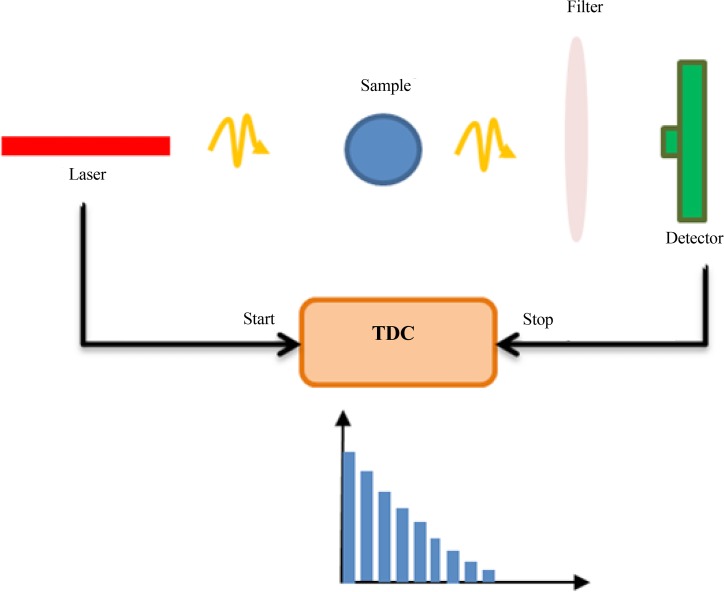
TCSPC setup which needs a laser source for light emission with electronic pulse output, some optics elements, a detector which can measure the photon arrival time accurately, and a time to digital converter, measuring the time difference between laser emission and photon detection.

Figure 5 shows the setup for the FLIM and TCSPC measurements. The setup uses a laser source which emits photons while simultaneously activates an electric pulse by each laser pulse emission. The sample, optics, and SPAD that is placed as detector comprise the other parts of the FLIM and TCSPC setup. The system needs a time to digital converter (TDC) to measure the difference between the photon emission and the photon detection. Some more digital circuits are used to create a histogram from the time and define the fluorescence lifetime.

**Figure 6:**
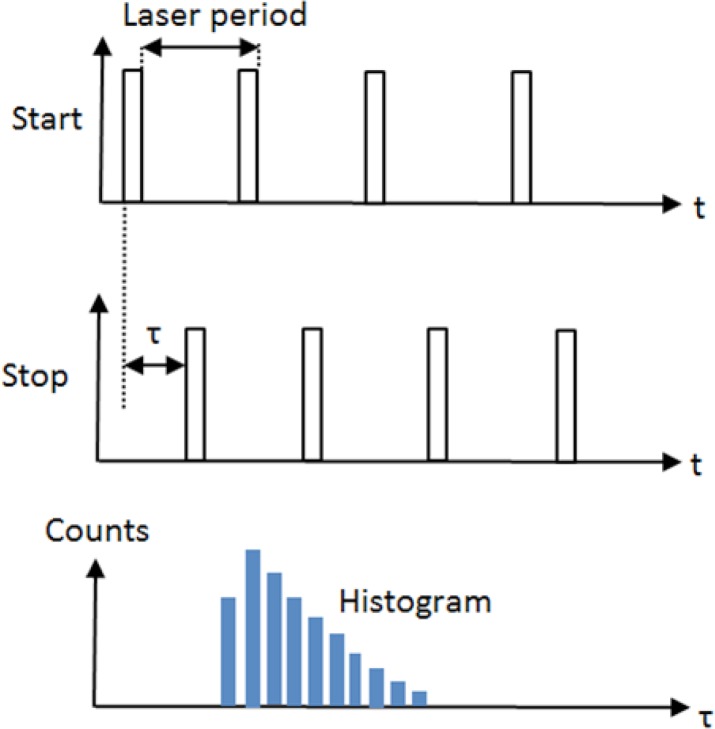
Start and the stop signal of TDC showing the photon emission and detection and the histogram of the time between start and stop signals. Start signal comes from the laser source and stop signals come from the detector.

Figure 6 shows the mechanism of the lifetime measurement. Two pulses are at the input of TDC named as start pulse, which the laser sends by emitting light and the stop signal, which detector sends at the photon detection time. The time difference between start and stop signals is measured and the histogram of the time difference is created in the processor memory. The florescence lifetime can be calculated through time difference histogram extraction.

## 3. Results

Several single-photon detectors are generally known for decades. The conventional devices are microchannel plates (MCPs) and photomultiplier tubes (PMTs) that are the sensors of choice in some applications (Karami, 2011). Even though PMTs have been evaluated since 1960s (Haitz, 1965), SPADs have recently become a serious competitor to MCPs and PMTs (Pavesi & Fauchet, 2008). SPADs have been proposed for imaging in applications where speed and or event timing accuracy are critical. Single-photon counters can be used in the above-mentioned processes for the neural monitoring.

**Figure 7:**
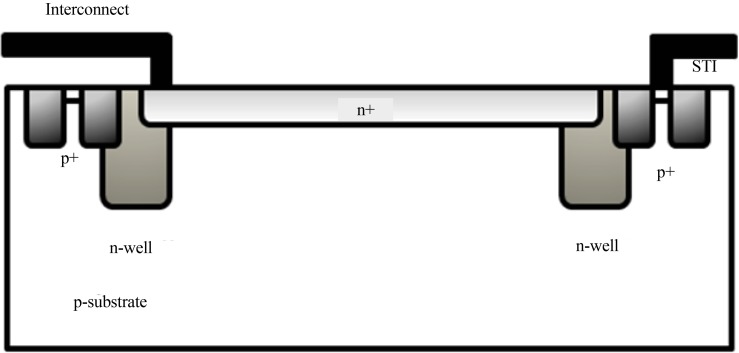
SPAD cross section implemented in (Karami, 2011). SPADs are easy to be implemented because of using standard CMOS technology for implementation and easy structure (diode) as the photosensitive area. STI is the shallow trench isolation (SiO_2_).

Figure 7 shows a SPAD cross section where the p-substrate n+ junction makes the photosensitive area. The n-wells are used as guard ring to prevent premature edge breakdown. Since SPADs are biased above the breakdown voltage, corners of the n+ layer can have highest electric field where the guard ring suppresses the high electric field and confines it to the photosensitive region.

SPADs can be implemented in standard and imaging CMOS technology, where silicon is used as the semiconductor substrate and billions of readout transistors can be implemented on the same chip.

**Figure 8:**
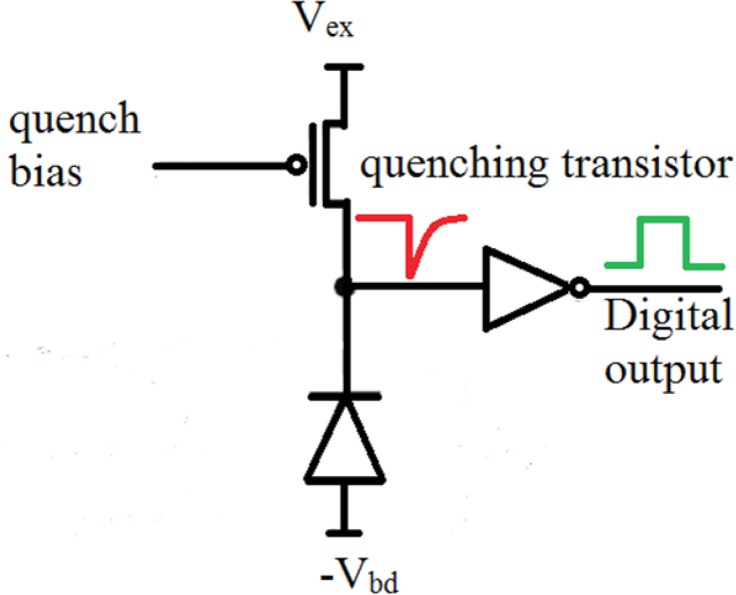
Passive quenching of SPAD current during avalanche. The quench transistor acts as a resistor to confine the diode (SPAD in this case) electric current and quench the avalanche current. V_bd_ stands for SPAD breakdown voltage, V_e_ is the amount of voltage which is based above the breakdown voltage.

SPADs are biased above the breakdown voltage in the so-called Geiger mode of operation. Figure 8 shows the circuit which is used to quench avalanche current generated by photon absorption. When a high current passes through SPAD, a high voltage will also drop on the quenching transistor and will lead to SPAD biased below the breakdown voltage to quench the avalanche. After the SPAD discharge, it is recharged to the biased condition after breakdown voltage and gets ready for the next photon detection. SPADs are characterized by some parameters to compare their effectiveness and performance. These parameters are listed below:

### 3.1. Breakdown voltage

SPAD is biased above the breakdown voltage to reach the Geiger mode of operation. Different SPADs, which are realized, have different breakdown voltages while the breakdown voltage is reducing with the CMOS technology advancement (Charbon, Yoon, & Maruyama, 2013). Since the breakdown voltage is a function of semiconductor doping profiles, which are increasing by the introduction of new CMOS technologies (Lundstorm & Guo, 2006), the breakdown voltage is scaling by the technology advancement.

The following Equation shows the relation between the breakdown voltage and the doping concentration (Pierret, 1996). where N_B_ is the doping concentration on the lightly doped side of the junction.

### 3.2. Dark count rate

Dark count rate (DCR) is the amount of spurious pulses from the SPAD in the complete dark. These pulses, which represent the noise factor and are not related to the light absorption, affect the responses of SPADs. While DCR due to the thermal generation is reducing by the technology advancement, the DCR due to band to band tunneling mechanism is increasing with the doping concentration surge.

$$V_{\mathrm{BR}}∼\frac{1}{{N^{0.75}}_{B}}$$

### 3.3. Photon detection probability

**Figure 9:**
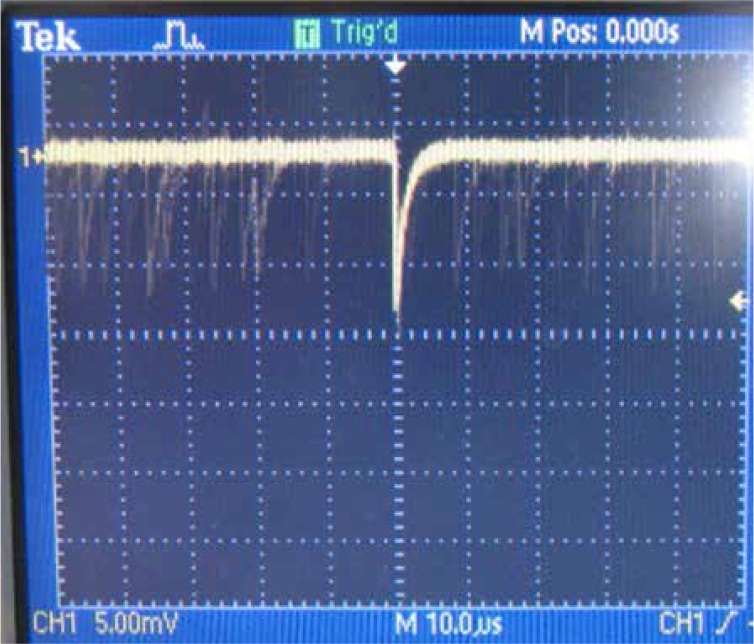
Passive quenching of the SPAD implemented in (Karami, 2011) during the avalanche. The quenching process leads to a temporal SPAD discharge, and then charging again for the next photon detection.

Photon detection probability (PDP) is a measure of how much light is detected and converted to electron-hole pairs. PDP is defined as the percentage of the total photons which are converted to the electrons. SPADs which are biased with a quenching transistor (Figure 9) generate a digital pulse with a photon arrival. The number of pulses divided by the number of photons emitted from laser to the SPAD is defined as the PDP.

### 3.4. Jitter

Jitter is the amount of timing uncertainty of photon detection. For example when a SPAD’s jitter is 200ps, it means the photon arrival can be estimated with 200ps error in the timing. Jitter is a function of CMOS technology where smaller depletion region of SPAD can lead to lower overall jitter (Karami, 2011).

### 3.5. Afterpulsing

Afterpulsing occurs since the SPADs generate correlated pulses due to the incomplete quench of the avalanche process or the generation of avalanche due to the existence of traps and defects in the substrate. Afterpulsing is characterized as a factor when correlated pulses are predicted without photon detection.

Table 1 shows the main characteristics of recent implemented SPADs. It should be noted that each SPAD pixel or camera is developed in a dedicated CMOS technology which is written in the last column of the table. Smaller technology can be translated as a more sophisticated technology, since making the transistors smaller is more difficult. Design and implementation in more sophisticated technologies are more difficult due to the introduction of quantum effects in the SPAD implementation (Karami, 2011).

**Table 1:** Recently realized SPADs’ figures of merit comparison.

**Reference**	**Breakdown Voltage (V)**	**DCR (kHz)**	**PDP (Peak)%**	**Afterpulsing Probability (%) @ Deadtime (ns)**	**Jitter (ps)**	**Technology Node (nm)**
(Mandai et al., 2012)	19.7	0.252–5.06	36(600 nm)	50@750	316	180
(Gersbach et al., 2009)	12.8	0.22	36(480 nm)	<1@180	128	130
(Richardson, Grant, & Henderson, 2009)	14.4	0.025	28(500 nm)	0.02@100	200	130
(Karami et al., 2010)	10.4	8.1	12(520 nm)	32@1200	398	90
(Webster et al., 2011)	14.9	0.1–10	44(690 nm)	0.375@n.a	84	90
(Karami, Amiri-Sani, & Ghormishi, 2014)	13.6	10	43(520 nm)	25@nominal	152	350
(Sun et al., 2013)	12.7	20–120	11.3	n.a	500	Silicon on insulator (SOI)

As Table 1 shows, the technology trend in SPAD production is to make lower DCR, higher PDP, lower jitter, and lower after pulsing SPADs. Progressing CMOS technology leads to lower breakdown voltages.

## 4. Discussion

SPADs can be used as the single-photon detector element for PET, SPECT, FLIM, and TCSPC. All mentioned imaging technologies are being used for the neural monitoring. Hence, design and implementation of SPADs with the minimum jitter, minimum DCR, minimum afterpulsing, and maximum PDP are needed for the application of neural imaging. Lower jitter SPADs can be translated to setup with higher speeds for the neural imaging process. While the implementation of low DCR SPADs in new deep submicron technologies is difficult due to the tunneling noise, the number of SPADs can be increased which results in higher resolution of imaging. Moreover, high PDP SPADs in the blue and green part of light can be used for high energy photon detection.
